# High Emotional Similarity Will Enhance the Face Memory and Face-Context Associative Memory

**DOI:** 10.3389/fpsyg.2022.877375

**Published:** 2022-05-09

**Authors:** Shu An, Mengyang Zhao, Feng Qin, Hongchi Zhang, Weibin Mao

**Affiliations:** School of Psychology, Shandong Normal University, Jinan, China

**Keywords:** facial expression, emotional similarity, emotional congruency, face memory, face-context associative memory

## Abstract

Previous research has explored how emotional valence (positive or negative) affected face-context associative memory, while little is known about how arousing stimuli that share the same valence but differ in emotionality are bound together and retained in memory. In this study, we manipulated the emotional similarity between the target face and the face associated with the context emotion (i.e., congruent, high similarity, and low similarity), and examined the effect of emotional similarity of negative emotion (i.e., disgust, anger, and fear) on face-context associative memory. Our results showed that the greater the emotional similarity between the faces, the better the face memory and face-context associative memory were. These findings suggest that the processing of facial expression and its associated context may benefit from taking into account the emotional similarity between the faces.

## Introduction

In daily life, when we encounter strangers, we often make decisions on whether to approach them by their appearance, especially by their facial expressions. Of course, facial expressions don’t appear in isolation, they are usually embedded in a rich and informative context (Wieser and Brosch, 2012). For instance, smiling facial expressions are more frequent at weddings, disgusted facial expressions seem to be paired with a garbage area, etc. Previous studies have examined the effect of the emotionality of contexts on the judgment of facial expressions (Barrett and Kensinger, 2010; Aviezer et al., 2011a; Barrett et al., 2011). Researchers found the judgment of facial expressions was facilitated in the emotionally congruent condition (i.e., the emotionality is congruent between facial expressions and their surrounding contexts), and facial expressions that were embedded in emotionally congruent scenes were categorized faster than that in incongruent scenes (Van den Stock et al., 2007, 2014; Righart and de Gelder, 2008a,b; Barrett and Kensinger, 2010; Aviezer et al., 2011a). Given the influence of emotional congruency between faces and contexts on the judgment of facial expressions, we were interested in how emotional congruency between faces and contexts would influence face memory and face-context associative memory.

Righi et al. (2015) were the first to use face-context composite pictures (happy faces or fearful faces that were superimposed on happy contexts or fearful contexts) to examine the item memory of emotional faces and the associative memory of face-context in the congruent and incongruent conditions. They found face memory benefited from the affective congruency between faces and contexts, with better memory for happy faces embedded in happy contexts. In contrast, although the associative memory of faces was not influenced by affective congruency between faces and contexts, the associative memory performance of happy faces was always higher than that of fearful faces. In the study of Righi et al. (2015), however, it is noteworthy that what researchers conducted was between-valence comparisons of faces (i.e., happy face vs. fearful face) rather than within-valence comparisons of faces (i.e., fearful face vs. angry face). Obviously, there are significant differences in facial configurations and perceptual similarity between happy faces and fearful faces (Aguado et al., 2018). Therefore, we speculated that when participants could easily distinguish the two kinds of facial expressions (e.g., happy face and fearful face), they might not rely on contexts to process faces, such that face-context associative memory did not benefit from the affective congruent condition. Furthermore, this also raised a question about what role the context played when facial expressions were hard to distinguish, especially when they were affectively congruent but emotionally incongruent (i.e., fearful face vs. angry face).

In fact, Aviezer et al. (2008) have ever proposed that the magnitude of contextual effect may be modulated by the degree of the emotional similarity presented in the facial expressions. For instance, disgusted faces are emotionally similar to angry faces but dissimilar to fearful faces. Therefore, disgusted faces will be strongly affected by angry contexts but weakly affected by fearful contexts. That is, people are more likely to perceive the disgusted faces embedded in the angry scenes as angry faces than perceive the disgusted faces embedded in the fearful scenes as fearful faces, which is a pattern of the similarity effect (Susskind et al., 2007; Aviezer et al., 2008, 2009). Further, Aviezer et al. (2011b) manipulated three different levels of similarity between the actually presented faces and the faces that are typically associated with the context emotion (i.e., emotionally congruent, high similarity, low similarity), and examined the effect of emotional similarity on face-context integration. Specifically, in the congruent condition, angry, disgusted, fearful, and sad faces appeared in their respective emotional context; in the high similarity condition, angry faces appeared in disgusted contexts, disgusted faces appeared in angry contexts, and sad faces appeared in fearful contexts; in the low similarity condition, angry faces appeared in sad contexts, sad faces appeared in angry contexts, and disgusted faces appeared in fearful contexts. Researchers found that participants were most likely to categorize the emotion of faces as the emotion of contexts in the congruent and high similarity conditions, while they were least likely to categorize the emotion of faces as the emotion of contexts in the low similarity condition. Such findings seemed to suggest that the greater the emotional similarity between the target face and the context-associated face was, the more it was for participants to rely on contexts to process faces.

Since the emotions of anger, disgust, and fear allow people to prioritize the detection and processing of important information, and keep the individual away from a potentially threatening environment (Rozin and Fallon, 1987), we aimed to further explore how emotional similarity of negative facial expressions would affect face memory and face-context associative memory. Our main interest was to test whether emotional congruency and high similarity contributed to enhancing face-context associative memory. Because it was difficult to distinguish between two highly similar faces, participants might attempt to use available context information to process the face. Especially, they might devote more processing resources to encoding and remembering the context when they were asked to perceive the emotion in a face (Barrett and Kensinger, 2010; Aviezer et al., 2011b). Therefore, we hypothesized that the higher the degree of emotional similarity between the target face and the face associated with the context emotion, the more likely participants were to rely on context information to process the target face, such that the better the associative memory between the target face and its paired context was. In other words, we speculated that the associative memory performance might be the greatest in the congruent condition, intermediate in the high similarity condition, and worst in the low similarity condition. Besides, given that facial expression processing might benefit from the emotional congruency between faces and contexts (Righi et al., 2015), we expected a better face memory in the emotionally congruent condition.

## Method

### Participants

Thirty-eight healthy undergraduates (11 males), aged 18–22 (*M* = 20.03, *SD* = 0.81) participated in this experiment. Data from one participant was discarded because the mean accuracy of most conditions was below 3 standard deviations from the mean. The remaining 37 participants had normal or corrected-to-normal vision. All of them were volunteers and received gifts after this experiment. Based on the effect size for the interaction between expression valence and context valence (*f* = 0.59) in the study of Righi et al. (2015), we set a medium effect size *f* = 0.25. According to G*power 3.1, the current sample size was adequate to achieve 95% power to observe an interaction between emotional face and emotional context (α = 0.05).

### Materials

#### Faces and Contexts

According to Aviezer et al. (2008), we used three kinds of negative facial expressions that were affectively congruent but emotionally incongruent (i.e., disgusted vs. angry vs. fearful) as experimental materials. We chose the images of facial expressions consisting of 60 disgusted faces, 50 angry faces, and 50 fearful faces from the Chinese facial affective picture system (Gong et al., 2011), 100 disgusted contexts, 70 angry contexts, and 70 fearful contexts from International Affective Picture System (IAPS; Lang et al., 1999). Half of the faces were males, and the other half were females. All the face images were gray-scale and were adjusted to the same size (130 × 148 pixels), luminance and contrast by using Photoshop CS6. All the context pictures were adjusted to the same size (700 × 525 pixels) by using Photoshop CS6.

Ten participants who did not participate in the formal experiment were asked to rate a series of dimensions of facial expressions and emotional contexts, respectively. Valence was rated on a 9-point scale (1 = the most negative, 9 = the most positive), arousal was rated on a 9-point scale (1 = extremely calm, 9 = extremely arousing), whether the label of facial expressions or contexts corresponding to one of the three basic emotions (disgusted, angry, and fearful) was rated on a 7-point scale (1 = not at all, 7 = very much) (Adolphs et al., 1994; Susskind et al., 2007). At last, 45 disgusted faces (mean valence = 3.67, *SD* = 0.78; mean arousal = 5.89, *SD* = 0.43), 45 angry faces (mean valence = 3.08, *SD* = 0.48; mean arousal = 6.79, *SD* = 0.33), and 45 fearful faces (mean valence = 3.19, *SD* = 0.80; mean arousal = 6.90, *SD* = 0.68) were selected. The label of these facial expressions was considered a good match to their actual emotion with a score above 6. Meanwhile, 45 disgusted contexts (mean valence = 3.33, *SD* = 0.82; mean arousal = 6.20, *SD* = 0.74), 45 angry contexts (mean valence = 3.15, *SD* = 0.41; mean arousal = 6.17, *SD* = 0.36), and 45 fearful contexts (mean valence = 3.29, *SD* = 0.97; mean arousal = 5.79, *SD* = 0.88) were selected. The label of these contexts was considered a good match to their actual emotion with a score above 6.

#### Face-Context Composite Pictures

Ninety faces (30 disgusted faces, 30 angry faces, 30 fearful faces) and 90 contexts (30 disgusted contexts, 30 angry contexts, 30 fearful contexts) were combined into 90 composite pictures as the materials in the study phase. These composite pictures thus produced three different levels of emotional similarity between faces and contexts: emotionally congruent, high similarity, and low similarity. Previous work has established that the facial expression of disgust shares a decreasing degree of emotional similarity with the facial expression of anger, sadness, and fear in order (Susskind et al., 2007; Aviezer et al. 2008, 2011b). Hence, in the congruent condition, disgusted faces, angry faces, and fearful faces appeared in their respective emotional context (e.g., a fearful face was placed onto a fearful context). In the high similarity condition, disgusted faces appeared in angry contexts, angry faces appeared in disgusted contexts. In the low similarity condition, disgusted faces appeared in fearful contexts, fearful faces appeared in disgusted contexts, angry faces appeared in fearful contexts, and fearful faces appeared in angry contexts. To ensure that essential parts of the context were not concealed, we placed half of the emotional faces on the left of the context and the other half on the right of the context.

Test materials consisted of 90 face images (45 studied face images and 45 new face images) and 135 context pictures (45 studied intact contexts that were presented with the 45 studied faces in the study phase, 45 studied rearranged contexts that were presented with the other 45 face images in the study phase, and 45 new ones that did not appear in the study phase).

### Procedure

In the study phase, participants were informed that they would view a series of composite pictures on the computer screen and needed to indicate the emotion evoked by the facial expression from a list of emotion labels (disgust, anger, fear, sadness, and happiness) listed under each picture. Meanwhile, they were asked to try their best to remember these pictures for a later memory test. After a fixation cross of 1,500 ms, 90 face-context composite pictures were presented for 5,000 ms one by one, including 30 emotionally congruent face-context composites (10 disgusted faces-disgusted contexts, 10 angry faces-angry contexts, 10 fearful faces-fearful contexts), 20 high similarity face-context composites (10 disgusted faces-angry contexts, 10 angry faces-disgusted contexts), and 40 low similarity face-context composites (10 disgusted faces-fearful contexts, 10 fearful faces-disgusted contexts, 10 angry faces-fearful contexts, 10 fearful faces-angry contexts). These composite pictures were randomly presented. Meanwhile, the same face that appeared in different emotional contexts was counterbalanced across participants, such that the same face was embedded either in a disgusted context, in an angry context, or in a fearful context to different participants.

After the study phase, participants completed a 5-min arithmetic task. They were asked to compute the sum of three-digit numbers.

**FIGURE 1:**
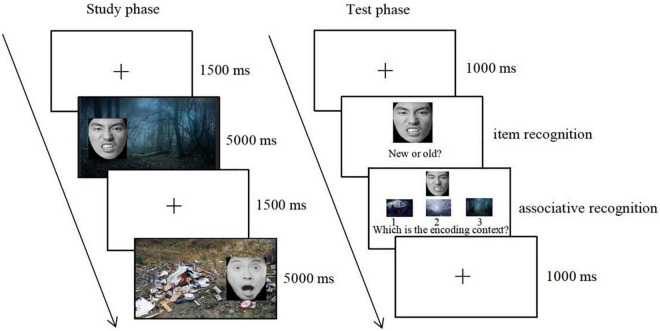
The procedure for the study phase and the test phase. Copyright: Chinese Facial Affective Picture System (Gong et al., 2011), republished with permission.

In the test phase, after a fixation cross was presented for 1,000 ms, 45 studied face images mixed with 45 new face images were presented randomly in the center of the screen. Participants were first asked to perform a self-paced recognition task and indicated whether they had seen the face during the study phase by pressing “F” (yes) or “J” (no). If participants correctly judged the studied face as “old” items, they were then shown the three context pictures. One was an “intact” context that was presented with the old face in the study phase, the second was a “recombined” context that was presented with a different face in the study phase, and the third was a “new” context that did not appear in the study phase. It should be noted that the “recombined” and “new” context were from the same emotional category as the “intact” context. Participants were required to make a decision on which context had been originally presented with the old face. The spatial position of the three contexts was counterbalanced across participants (see Figure 1).

### Data Analysis

No trial was excluded from the analysis. According to Bisby and Burgess (2014) and Funk and Hupbach (2014), item memory and associative memory scores were corrected by hits minus false alarms. Since the violations of normality for most conditions, all data were analyzed using non-parametric tests (Friedman test and Wilcoxon signed rank test). Bonferroni correction was used for multiple pairwise comparisons.

## Results

### Item Memory (Face Memory)

**FIGURE 2:**
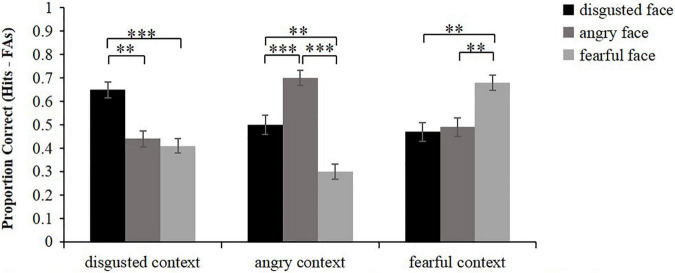
Mean proportion correct for face recognition scores (hits minus false alarms) as a function of emotional face and emotional context. Error bars represent standard error. ^**^p < 0.01, ^***^p < 0.001.

The corrected accuracy of face memory was calculated as hits (old faces were correctly recognized as being old) minus false alarms (new faces were incorrectly recognized as being old) (Figure 2). Friedman test was conducted to analyze the corrected recognition scores of faces. The results showed that there were significant differences in item memory among different conditions, *χ^2^*(8) = 86.14, *p* < 0.001. Wilcoxon signed rank test was then used to conduct pairwise comparisons. The results showed that for the faces that were embedded in disgusted contexts, disgusted faces were remembered better than angry faces and fearful faces (*z* = 3.36, *p* = 0.003; *z* = 3.94, *p* < 0.001), but the memory performance of angry faces did not differ from that of fearful faces (*z* = 0.67, *p* = 1.000). For the faces that were embedded in angry contexts, angry faces were remembered better than disgusted faces and fearful faces (*z* = 3.63, *p* < 0.001; *z* = 4.96, *p* < 0.001), and disgusted faces were remembered better than fearful faces (*z* = 3.10, *p* = 0.006). For the faces that were embedded in fearful contexts, fearful faces were remembered better than disgusted faces and angry faces (*z* = 3.21, *p* = 0.003; *z* = 3.23, *p* = 0.003), while the memory performance of disgusted faces did not differ from that of angry faces (*z* = 0.12, *p* = 1.000). In brief, these results basically revealed that face memory was always the best in the congruent condition. Meanwhile, face memory of the high similarity condition was greater than that of the low similarity condition when faces were embedded in angry contexts.

### Associative Memory (Memory for the Intact Contexts Associated With Faces)

**FIGURE 3:**
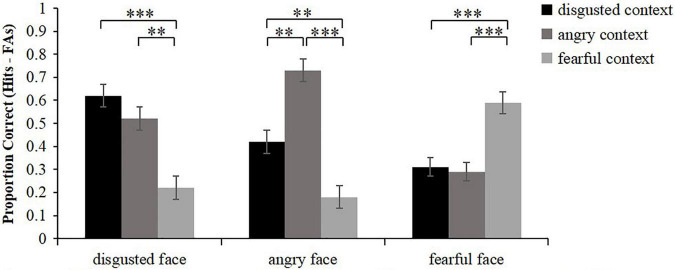
Mean proportion correct for recognition of the associated intact context (hits minus false alarms) as a function of emotional face and emotional context. Error bars represent standard error. ^**^p < 0.01, ^***^p < 0.001.

Similarly, the corrected accuracy for associative memory was calculated as hits (intact contexts were correctly recognized as being intact) minus false alarms (new contexts and recombined contexts were incorrectly recognized as being intact) (Figure 3). Friedman test was conducted to analyze the corrected recognition scores of intact contexts. The results showed that there were significant differences in associative memory among different conditions, *χ^2^*(8) = 86.36, *p* < 0.001. Wilcoxon signed rank test was then used to conduct pairwise comparisons. The results showed that for the intact contexts that were associated with disgusted faces, disgusted intact contexts and angry intact contexts were both remembered better than fearful intact contexts (*z* = 4.21, *p* < 0.001; *z* = 3.48, *p* = 0.003), while the memory performance of disgusted intact contexts did not differ from that of angry intact contexts (*z* = 1.32, *p* = 0.564). For the intact contexts that were associated with angry faces, angry intact contexts were remembered better than disgusted intact contexts and fearful intact contexts (*z* = 3.42, *p* = 0.003; *z* = 4.65, *p* < 0.001), and the memory performance of disgusted intact was greater than that of fearful intact contexts (*z* = 3.11, *p* = 0.006). For the intact contexts that were associated with fearful faces, fearful intact contexts were remembered better than disgusted intact contexts and angry intact contexts (*z* = 3.55, *p* < 0.001; *z* = 3.74, *p* < 0.001), while the memory performance of disgusted intact contexts did not differ from that of angry intact contexts (*z* = 0.22, *p* = 1.000). In sum, these results revealed that the associative memory of disgusted faces was better in the congruent condition and high similarity condition than that in the low similarity condition. The associative memory of angry faces was the best in the congruent condition, intermediate in the high similarity condition, and worst in the low similarity condition. Meanwhile, the associative memory of fearful faces was better in the congruent condition than that in the low similarity condition.

## Discussion

On the basis of the prior work concerning the effect of emotional similarity on the judgment of facial expression (Susskind et al., 2007; Aviezer et al., 2008, 2011b), we established three kinds of emotional similarity conditions (i.e., emotionally congruent, high similarity, low similarity) to examine the effect of emotional similarity of negative facial expressions on face memory and face-context associative memory. As predicted, our results indicated that the face memory and the face-context associative memory were the greatest in the congruent condition, intermediate in the high similarity condition, and worst in the low similarity condition as a whole. These similarity patterns not only extended the findings obtained from previous studies, but also contributed to understanding face-context integration process.

First, face memory and face-context associative memory both benefited from emotional congruency and high similarity. The results from item memory showed face memory performance was the highest in the emotionally congruent context, intermediate in the high similarity context, and worst in the low similarity context, suggesting that the processing of facial expressions could be strongly influenced by contexts, especially when the target face was emotionally similar to the facial expression associated with the context (Aviezer et al., 2008, 2011b). Such results were also in line with previous studies that showed the advantage of the emotional congruency effect. For instance, facial expressions were recognized faster or were remembered better in emotionally congruent contexts than in incongruent contexts (Righart and de Gelder, 2008a,b; Diéguez-Risco et al., 2013, 2015; Righi et al., 2015).

Meanwhile, the results from associative memory were somewhat consistent with the pattern observed in item memory, showing that face-context associative memory was the best in the emotionally congruent context, intermediate in the high similarity context, and worst in the low similarity context. In fact, Barrett and Kensinger (2010) have ever reported that perceivers might encode the context when they were required to make a more specific inference about a target person’s emotion. Therefore, we speculated that when two highly similar faces were presented, it was difficult for participants to perceive the emotion of faces just by the face itself. In such a case, participants might use whatever they could to distinguish between highly similar faces, such as available context information. As a consequence, they might devote more attention to encoding and remembering the context, such that face-context associative memory was enhanced in the congruent and high similarity conditions.

Second, face-context associative memory benefited from the congruent contexts and high similarity contexts, which might potentially be explained by unitization. A substantial body of research has shown that when two distinct items were processed as a single unitized item, such as the word pair “traffic-jam,” the associative memory of items would be enhanced (Quamme et al., 2007; Parks and Yonelinas, 2009, 2015). Because the intact contexts associated with faces shared more emotional characteristics with faces and were remembered better in the congruent and high similarity conditions, we speculated that faces and contexts were more likely to be unitized as a coherent whole in congruent and high similarity conditions compared to low similarity condition. In other words, emotional congruency and high similarity might be conducive to promoting the level of unitization between faces and contexts, thereby increasing face-context associative memory. However, it is noteworthy that more research is needed to test whether high emotional similarity would be a way of promoting unitization between items.

Third, it is important to note that face-context associative memory would be enhanced when the target face and the face associated with the context emotion were highly emotionally similar. Righi et al. (2015) investigated how different emotional valence (positive or negative) influenced face memory and face-context associative memory. And they found that happy and fearful emotions might influence the binding process between faces and contexts in different ways. Namely, regardless of whether the context was positive or negative, positive faces might broaden the attention and foster the integration of faces and surrounding contexts (Shimamura et al., 2006; Tsukiura and Cabeza, 2008), while negative faces might narrow attention and impair the encoding of surrounding contexts associated with faces (Mather, 2007; Mather and Sutherland, 2011; Murray and Kensinger, 2013). By contrast, in the present study, we manipulated the emotional similarity of negative faces, and found that negative emotion also could promote face-context associative memory when two negative faces were highly emotionally similar. Of course, given that the emotions of disgust, anger, and fear are likely to be processed with partially different neural systems of the brain (Calder et al., 2001, 2004; Anderson et al., 2003), it may be interesting to further study whether these brain regions are involved in processing emotional faces and contexts, and whether the neural activities would be enhanced when the emotionality between facial expressions and surrounding contexts is congruent or highly similar.

It is important to point out that in this study, we only focused on the emotional similarity of negative facial expressions. More research is still needed to understand the relationship between emotional similarity (e.g., emotional similarity of positive facial expressions) and memory. Besides, although the facial expressions we used were all negative, there were some differences in the arousal among different facial expressions. The differences of arousal might affect memory performance, which should be controlled for in future studies.

Taken together, our findings suggest that the greater the emotional similarity between the faces, the better the face memory and face-context associative memory were. The present results not only enrich the theoretical knowledge about the effect of emotional similarity on associative memory, but may also have important implications for understanding how and to what extent the interplay between facial expression and emotional context can affect an individual’s memory in everyday social interactions.

## Data Availability Statement

The raw data supporting the conclusions of this article will be made available by the authors, without undue reservation.

## Ethics Statement

The studies involving human participants were reviewed and approved by the Academic Board of Shandong Normal University. The patients/participants provided their written informed consent to participate in this study. Written informed consent was obtained from the individual(s) for the publication of any potentially identifiable images or data included in this article.

## Author Contributions

WM conceived and designed the experiments, and wrote the manuscript with SA and MZ. FQ performed the experiments and collected the data. SA and HZ analyzed the data under the supervision of WM. All authors contributed to the article and approved the submitted version.

## Conflict of Interest

The authors declare that the research was conducted in the absence of any commercial or financial relationships that could be construed as a potential conflict of interest.

## Publisher’s Note

All claims expressed in this article are solely those of the authors and do not necessarily represent those of their affiliated organizations, or those of the publisher, the editors and the reviewers. Any product that may be evaluated in this article, or claim that may be made by its manufacturer, is not guaranteed or endorsed by the publisher.
